# Discrimination, Allostatic Load, and Cognition among Indigenous, Black, Hispanic, and White Older Adults in the HRS

**DOI:** 10.1093/geroni/igaf122.1161

**Published:** 2025-12-31

**Authors:** Cliff Whetung, Ernest Gonzales

**Affiliations:** University of Minnesota Duluth, Duluth, Minnesota, United States; New York University, New York, New York, United States

## Abstract

The number of Indigenous older adults (IOAs) will more than double in the next thirty years. IOAs manifest dementia more often, and earlier in life than non-Hispanic Whites. Despite this, they remain underrepresented in cognitive health research. This study examined whether associations between perceived discrimination experiences (everyday discrimination, major lifetime discrimination) and cognition were mediated by allostatic load (AL), a measure of physiological stress. We used eight years (2008-2016) of restricted Health and Retirement Study data to examine cognitive trajectories among Indigenous (n = 222), Black (n = 2,181), Hispanic (n = 1,527) and White (n = 15,007) older adults using mixed effect growth curves. Regression models were adjusted for theoretically informed covariates. Both Indigenous and Black older adults reported frequent discrimination experiences and high standardized AL scores relative to non-Hispanic Whites. In regression analysis, we found that AL partially mediated the relationship between discriminatory experiences and cognition in minimally adjusted models, but this relationship lost statistical significance in fully adjusted models. Indigenous older adults have a unique profile that places them at elevated risk for cognitive pathology in later life, and structural inequities are powerfully associated with many of these risk factors. While these risk factors were associated with higher allostatic loads, our algorithm of AL did mediate the association between these risk factors and cognition. This finding conflicts with existing minority stress hypotheses, but may be the product of AL measurement issues. We conclude that dementia research integrating measures of AL requires additional transparency on the sensitivity and reliability of the construct.

